# Radial extracorporeal shock wave treatment harms developing chicken embryos

**DOI:** 10.1038/srep08281

**Published:** 2015-02-06

**Authors:** Maren C. Kiessling, Stefan Milz, Hans-Georg Frank, Rüdiger Korbel, Christoph Schmitz

**Affiliations:** 1 Extracorporeal Shock Wave Research Unit, Department of Anatomy II, Ludwig-Maximilians-University of Munich, Munich, Germany; 2 Clinic for Birds, Reptiles, Amphibians and Pet Fish, Ludwig-Maximilians-University of Munich, Oberschleissheim, Germany

## Abstract

Radial extracorporeal shock wave treatment (rESWT) has became one of the best investigated treatment modalities for cellulite, including the abdomen as a treatment site. Notably, pregnancy is considered a contraindication for rESWT, and concerns have been raised about possible harm to the embryo when a woman treated with rESWT for cellulite is not aware of her pregnancy. Here we tested the hypothesis that rESWT may cause serious physical harm to embryos. To this end, chicken embryos were exposed *in ovo* to various doses of radial shock waves on either day 3 or day 4 of development, resembling the developmental stage of four- to six-week-old human embryos. We found a dose-dependent increase in the number of embryos that died after radial shock wave exposure on either day 3 or day 4 of development. Among the embryos that survived the shock wave exposure a few showed severe congenital defects such as missing eyes. Evidently, our data cannot directly be used to draw conclusions about potential harm to the embryo of a pregnant woman treated for cellulite with rESWT. However, to avoid any risks we strongly recommend applying radial shock waves in the treatment of cellulite only if a pregnancy is ruled out.

Extracorporeal shock waves (ESW) are single acoustic high-pressure pulses generated by electrohydraulic, electromagnetic, piezoelectric, or ballistic/radial methods^1^,^2^,^3^. They are extensively used for treating kidney stones (extracorporeal shock wave lithotripsy; ESWL)^4^,^5^, various conditions of the musculoskeletal system^6^,^7^, and soft tissue wounds^8^. Radial extracorporeal shock waves (rESW) are generated by accelerating a projectile within a guiding tube. When the projectile strikes a metal applicator at the end of the guiding tube a stress wave is generated in the applicator. This stress wave is then transmitted as a radial shock wave into tissue^7^. Radial extracorporeal shock waves differ from focused extracorporeal shock waves (fESW) by penetration depth and certain physical properties. Specifically, the maximum energy flux density (EFD) of rESW is reached at the tip of the applicator, whereas the maximum EFD of fESW is reached in a focus zone within the treated tissue (Fig. 1).

Some fESW devices^9^,^10^ generate pulses that fulfill the characteristics set out by the physical definition of “true” shock waves^11^, whereas other fESW devices^7^,^12^ and rESW devices^7^,^10^ do not. However, both rESW and fESW are characterized by an initial high positive peak pressure between 10 and 100 Megapascal (MPa) reached in less than one microsecond (µs), a low tensile amplitude (negative pressure) following the positive pressure amplitude, a short life cycle of approximately 10–20 µs, and a broad frequency spectrum^7^,^9^,^10^,^12^. During the tensile phase cavitation, the formation of vapour bubbles of a liquid in a region where the pressure of the liquid falls below its vapor pressure^1^, may occur for both radial and focused shock waves^7^,^9^,^11^. These vacuum bubbles typically induce local shear forces when collapsing at the end of the phase of negative pressure^11^. Shock waves have both a direct effect (the result of the transferred energy) and an indirect effect (the result of the creation of cavitation) on the targeted tissue, and thus it has been hypothesized that both the direct and indirect effects produce a biological response in the treated tissue^1^,^7^,^11^.

In recent years radial extracorporeal shock wave treatment (rESWT) has also became one of the best investigated treatment modalities for cellulite^13^,^14^,^15^, including the abdomen as a treatment site^16^. Notably, pregnancy is considered a contraindication for rESWT, mostly based on earlier reports about potential damaging effects of fESW on embryos^17^,^18^,^19^,^20^,^21^. Accordingly, concerns have been raised about possible harm to the embryo when a woman treated with rESWT for cellulite is not aware of her pregnancy. This cannot be tested on pregnant mice or rats because rESW could rupture the gut of the dam^22^. We therefore exposed chicken embryos *in ovo* to various doses of rESW on either day 3 or day 4 of development (as done earlier with fESW^17^). Key features of chicken development by these time-points are a well developed circulatory system, an identifiable telencephalon, well established primary optic vesicles, and the formation of appendages^23^. This resembles the developmental stage of four- to six-week-old human embryos^24^.

## Results

**Figure 2:**
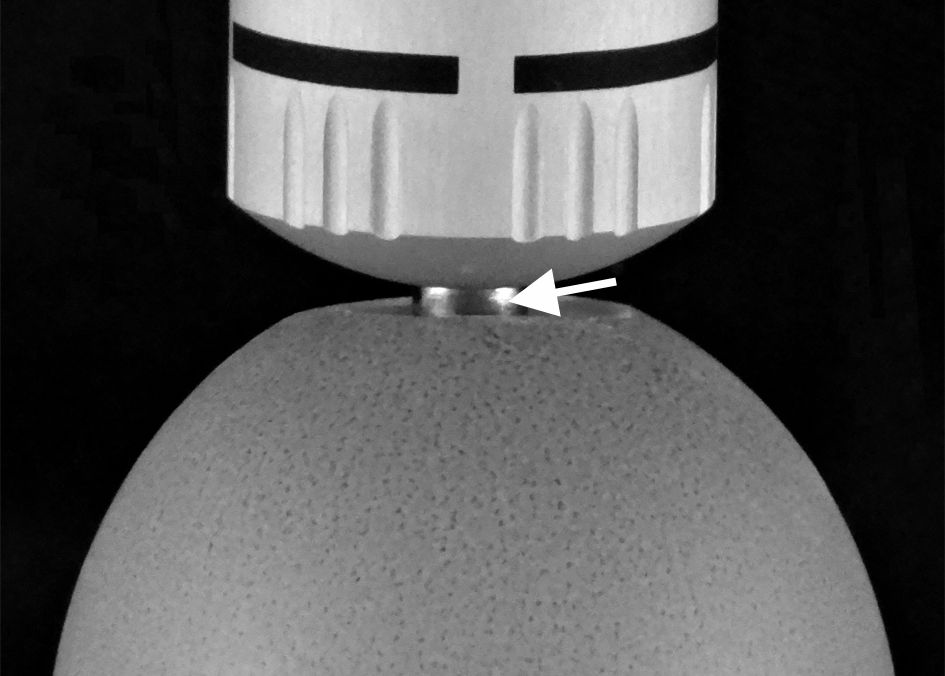
Exposure of fertilized Leghorn chicken eggs with radial extracorporeal shock waves. The applicator (arrow) of the rESW device was positioned in a hole generated in the egg shell at the position of the air sack.

Radial extracorporeal shock waves were generated with the Swiss DolorClast device (Electro Medical Systems, Nyon, Switzerland), used for cellulite treatment^15^. The 6-mm applicator of the “radial” handpiece of the device was placed in an opening generated in the egg shell of fertilized Leghorn chicken eggs at the position of the air sack (Fig. 2). Then each egg was exposed to a number of radial shock waves (between 0 and 75), generated at 3.8 bar air pressure of the shock wave device and 5 Hz frequency. The egg shell opening was then covered, eggs were kept in an incubator at 37.8°C and 58% humidity with 6 turns per day, and development of the embryos was repeatedly monitored using an egg candler. Eggs with indications of embryo death were opened immediately; otherwise eggs were opened on day 16 of development and embryos were sacrificed and photographed.

**Figure 3:**
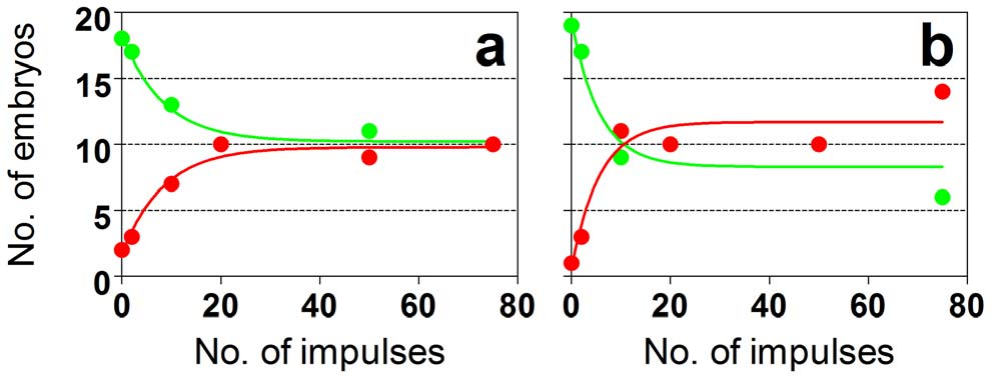
Number of intact (green dots) and malformed and/or dead (red dots) chicken embryos as a function of the number of applied radial shock waves on day 3 (a) or day 4 (b) of development.

We found a dose-dependent decrease in the number of surviving embryos after shock wave exposure on either day 3 or day 4 of development (Fig. 3). Statistical analysis of the relation between the number of surviving embryos and the number of applied shock waves using the one phase decay model showed a goodness of fit (R^2^) of respectively 0.969 and 0.902 for the shock wave exposure on either day 3 or day 4 of development, indicating very good fit of the experimental data and the model.

**Figure 4:**
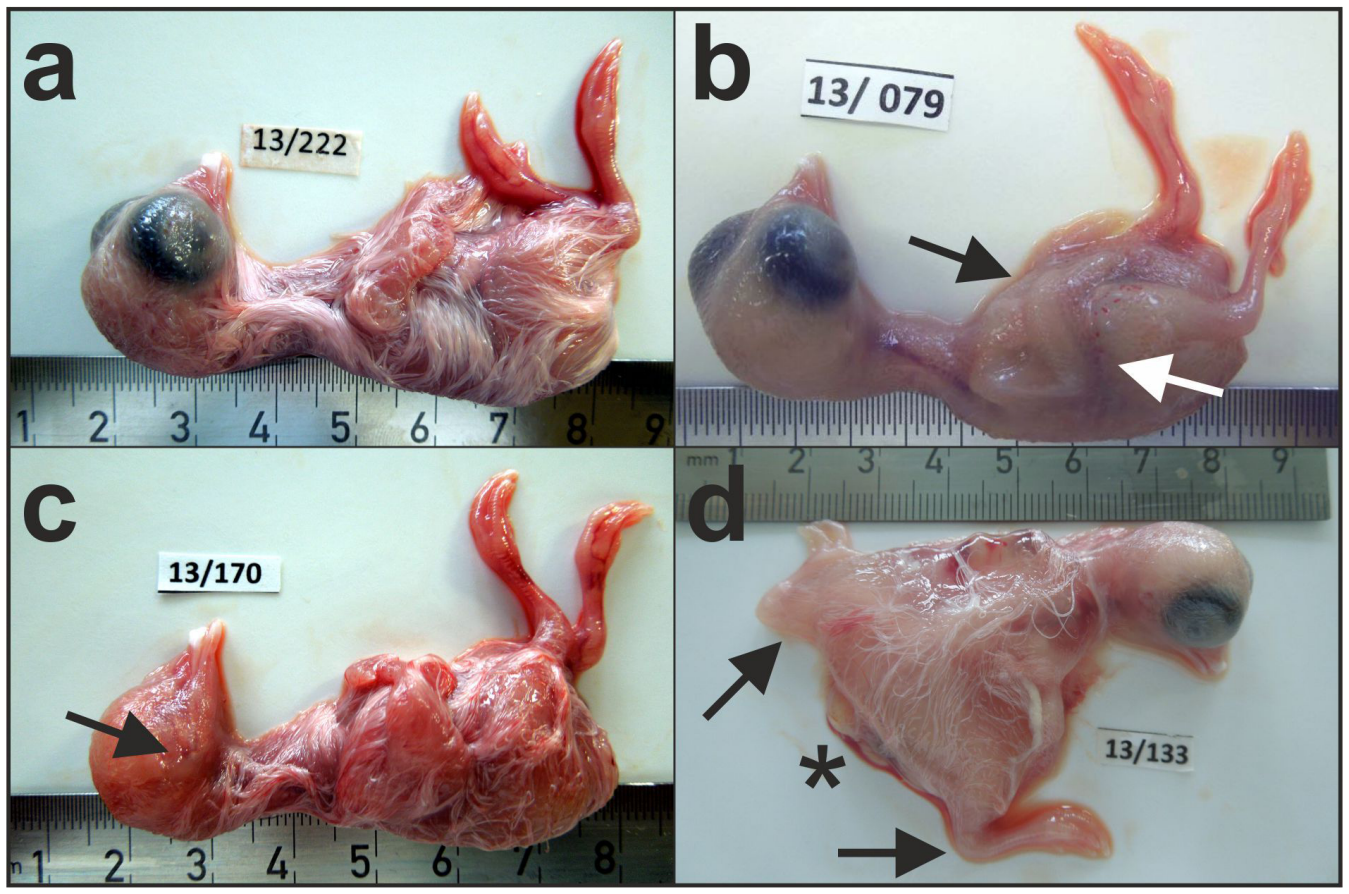
Example of chicken embryos sacrificed on day 16 of development. (a) Embryo not exposed to rESW. (b) Malformed embryo, exposed on day 3 with 10 pulses. The arrows points to the missing coat. (c) Malformed embryo, exposed on day 3 with 20 pulses. The arrow points to the missing eye. (d) Malformed embryo, exposed on day 4 with 50 pulses. The asterisk indicates the malformed pelvis, forcing the legs into a massive abduction position (arrows).

Among the 153 out of 240 embryos that survived the shock wave exposure, three showed severe congenital defects (missing eyes, missing coat or malformed pelvis) (Fig. 4).

## Discussion

This is the first report about serious physical harm to embryos exposed to rESW. Comparable, albeit mixed results, have been published over the last 25 years for fESW used in ESWL. For example, Hartman et al. (1990)^17^ incubated chicken embryos for three days before applying a total of three fESW to each embryo through an opening in the egg shell (shock waves were generated with a 9 French probe of a Wolf Model 2137.50 electrohydraulic lithotripter; Richard Wolf, Knittlingen, Germany). The authors found massive embryotoxic and teratogenic effects such as malformations and embryonic death in different stages of development (similar to the results reported in this study). However, Hartman et al. (1990)^17^ provided no details about malformations. Smith et al. (1992)^18^ exposed rats during early pregnancy to fESW generated with a Dornier Multipurpose Lithotripter (MPL 9000; Dornier MedTech, Wessling, Germany) and found weight retardations in the fetuses compared to unexposed controls. Frankenschmidt and Heisler (1998)^19^ exposed fetuses of 30 gravid rabbits to fESW on day 20 or 25 of gestation under technical conditions corresponding to ESWL in humans (2000 impulses at 75 MPa focal pressure per rabbit generated with a Piezolith 2300 lithotripter; Richard Wolf). Damaging effects on fetuses were examined by abdominal section 24 hours or 9 days later. Shock wave targeting of the cranium, thorax, abdomen, or placenta was usually lethal to the fetuses. When the uterine wall or the space between two fetuses was targeted, the fetuses suffered from superficial hematoma. When the distance between the fetuses and the midpoint of the focus zone was more than 1.5 cm fetuses were vital and free of lesions. In another experiment Frankenschmidt and Heisler (1998)^19^ exposed the kidneys of pregnant rabbits on day 11 of gestation to the same shock wave treatment as in their first experiment (outlined above). The authors found no statistically significant embryotoxic or teratogenic effects, neither from maternal data (resorption) nor from fetal findings (body measurements, vitality test, inner organs, skeletal deformities). Frankenschmidt and Heisler (1998)^19^ concluded that embryotoxic or teratogenic sequelae do not occur when shock waves are focused outside the uterus. In line with this, Bayrak et al. (2009)^20^ found no histomorphological changes in ovarian tissue when exposing the distal ureteral segment of rabbits to fESW (1500 impulses per rabbit generated with a Multimed 200 lithotripter [Elmed, Turkey] operated at 17 kV). The latter results supported data from a retrospective clinical survey by Vieweg et al. (1992)^21^ who concluded that ESWL of lower ureteral calculi is a safe and effective procedure, and does not affect female fertility or lead to increased teratogenic risk. However, it should be noted that the latter study was motivated by a spontaneous abortion in a young woman unaware of her pregnancy 24 hours after ESWL of a distal ureteral calculus^21^.

In summary, both rESW and fESW may seriously harm embryos. The underlying molecular and cellular mechanisms are completely unknown, and may result from complex interactions of tissues with compressional and shear stresses during the positive phase of the wave propagation as well as cavitation from the negative phase of the wave propagation^1^,^7^,^11^.

Evidently, our data cannot directly be used to draw conclusions about potential harm to the embryo of a pregnant woman treated for cellulite with rESW. However, considering that rESW could reach the human embryo, to avoid any risks we strongly recommend applying rESW in the treatment of cellulite only if a pregnancy is ruled out. Trivializing rESWT as “vibrating massage therapy” for cellulite by some authors^14^ is unjustified and may cause serious harm.

## Methods

All experiments were performed according to German animal protection regulations which do not require registration or approval of experiments with fertilized avian eggs.

### Eggs

Fertilized chicken eggs (Lohmann selected Leghorn; Lohmann, Cuxhaven, Germany) were received from the Clinic for Birds, Reptiles, Amphibians and Pet Fish at Ludwig-Maximilians-University of Munich (Oberschleissheim, Germany). The laying hens were kept under conditions specifically pathogen-free for avian adeno viruses group 1, avian encephalomyelitis virus, avian infectious bronchitis virus, avian infectious laryngotracheitis virus, avian leukosis viruses/antibodies subtypes a, b, j, avian leukosis viruses - p27 antigen, avian nephritis virus, avian orthoreoviruses, avian reticuloendotheliosis virus, avibacterium paragallinarum, chicken anemia virus (cav), egg drop syndrome virus, fowlpox virus, infectious bursal disease virus (ibdv) serotype 1 and serotype 2, influenza A virus, Marek's disease virus, turkey rhinotracheitis virus, newcastle disease virus, mycobacterium avium, mycoplasma gallisepticum, mycoplasma synoviae, salmonella pullorum, and salmonella spp.

Before breeding eggs were stored tip downwards in a fridge at 8°C for one to ten days. This position was kept all the time during breeding, treatment, and handling. Breeding was started by placing the eggs at room temperature for two hours. During this time their size (length, circumference) and weight were measured. Then, eggs were transferred into a rocking incubator (Top-Profi 180; J. Hemel Brutgeräte GmbH & Co. KG, Verl, Germany) and kept there at 37.8°C and 58% humidity with 6 turns per day (every four hours).

### Generation of shock waves

Radial shock waves were generated with a radial shock wave device, Swiss DolorClast (Electro Medical Systems, Nyon, Switzerland). The device generates radial shock waves by accelerating a projectile within a guiding tube with the aid of compressed air (maximum air pressure: 4.0 bar). When the projectile strikes a metal applicator at the end of the guiding tube a stress wave is generated in the applicator. This stress wave is then transmitted as a radial shock wave into tissue or in experimental settings into water.

### Application of shock waves

For dose-response analysis, six groups of eggs each were exposed to different shock wave doses (0, 2, 10, 20, 50 or 75 shock waves) on either day 3 or day 4 of development until n = 20 eggs per group with uninjured egg membrane were obtained. Shock wave exposure damaged the egg membrane of 41 eggs. Accordingly, a total of n = 281 eggs were used in the present study, and data from n = 240 eggs were included in the analysis.

Eggs were photographed and candled using an egg candler (OvaScope; Brinsea Product, Inc., Titusville, FL, USA) on the day of shock wave exposure. Unfertilized eggs (<10%) were discarded. Then an opening with a diameter of approximately 10 mm was generated in the egg shell at the site of the air sack using a mini saw (Power Plus X134; Varo - Vic.Van Rompuy nv, Lier, Belgium). The cavity was filled with 0.9% NaCl (Braun Melsungen AG; Melsungen; Germany).

For application of shock waves eggs were bedded on rice, and the “radial” handpiece of the Swiss DolorClast equipped with the 6-mm applicator was set vertically in a drill stand (Wolfcraft, Kempenich, Germany). The air pressure of the device was set to a constant 3.8 bar (yielding an energy flux density of the shock waves of 0.023 mJ/mm^2^ at a distance of 5 mm to the applicator; details are provided below) and the application frequency was set to 5 Hz. Then, the shock wave applicator was slowly lowered into the center of the hole in the egg shell and fixed in place once the tip of the applicator was immersed in the saline within the cavity in the egg. Upon confirmation of proper placement of the applicator within the saline the given number of shock waves was applied.

Following application, the handpiece was slowly raised from the egg and the applicator was cleaned before further usage to prevent sample contamination. The saline was removed from the cavity in the egg using a sterile 20-gauge needle (Sterican; Braun Melsungen), the opening generated in the egg shell was covered with Parafilm M (Pechiney Plastic Packaging, Chicago, IL, USA), and the eggs were put back into the incubator.

### Monitoring after shock wave exposure

Survival and development of the embryos was regularly controlled by candling the eggs for signs of life (such as movement and heartbeat) and development on different days after shock wave exposure. Eggs were opened and embryos investigated on day 16 of development. Eggs with indications of embryo death (such as cessation of development or decay scent) were opened earlier.

### Investigation of embryos at the end of the experiments

After cracking the egg shell the embryo was placed in a petri dish and photographed. Then, the embryo was transferred to a white tray and another photograph was taken. If the embryo did not die spontaneously, it was sacrificed by decapitation. Dead embryos were placed in 4.5 percent formalin solution (Carl Roth GmbH + Co. KG, Karlsruhe, Germany) and stored at room temperature.

### Statistical analysis

The relation between the number of surviving embryos and the number of applied shock waves was tested using best-fit nonlinear regression analysis, using the one-phase decay model. Calculations were performed using GraphPad Prism (version 5.00 for Windows, GraphPad Software, San Diego, CA, USA).

### Acoustic measurements using a laser fiber optic probe hydrophone

Measurements of the pressure field generated by the Swiss DolorClast with the “radial” handpiece equipped with the 6-mm applicator were carried out according to IEC-61846:1998 (Ultrasonics - Pressure pulse lithotripters - Characteristics of fields) in a 300 liter tank filled with demineralized water (conductivity approximately 5 µS/cm) at the laboratories of Electro Medical Systems (Nyon, Switzerland). The inner dimensions of the tank were 960 × 560 mm with a height of 660 mm. The water level rose to 470 mm when the applicators were immerged at a height of 330 mm.

Measurements were performed within an blown-out egg completely submerged under water with a laser hydrophone (FOPH 2000; RP Acoustics, Leutenbach, Germany) coupled to an oscilloscope (LeCroy 9361; LeCroy, Chestnut Ridge; NY) in the x-axis of the applicator. Positioning of the laser hydrophone probe was controlled with step motors, allowing a resolution of the position of 0.1 mm. Measurements were performed within the blown-out egg at a distance of 1, 2, …, 10, 12, 15, 20, 25, 30, 35, and 40 mm to the applicator while operating the Swiss DolorClast at 3.8 bar air pressure. All measurements were repeated five times and the results were averaged.

The electrical signal recorded by the oscilloscope was linked to the pressure signal (*P*) according to Equation 1: 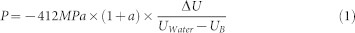
with *α* the reflection factor (given at 0.07), *ΔU* the measured electrical signal, *U_Water_* the reference voltage of the noise of the measurement (probe without laser), and *U_B_* the reference voltage of the probe (with laser activated).

The energy flux density (*J*) is the integral of the pressure as shown in Equation 2: 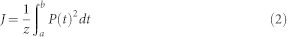
with *Z* the impedance of sound in water (1.5 × 10^6^ kg × m^−2^ × s^−1^), *P(t)* the pressure as a function of time, *a* the first positive extreme of the first measured pressure peak, and *b* the second positive extreme of the first measured pressure peak.

**Figure 5:**
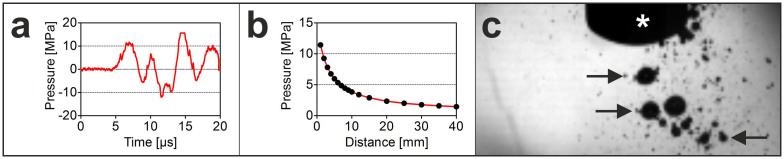
Characterization of the acoustic field generated with the radial extracorporeal shock wave device used in the present study. (a) Pressure as a function of time at a distance of 1 mm from the tip of the applicator. (b) Pressure as a function of the distance from the tip of the applicator (note that measurements shown in [a,b] were performed within an blown-out egg completely submerged under water using a laser fiber optic probe hydrophone). (c) Cavitation bubbles (arrows) generated during the phase of negative pressure of the radial extracorporeal shock waves, detected with a high-speed CCD camera.

Results were graphically represented using GraphPad Prism software (version 5; GraphPad, San Diego, USA) (Fig. 5a,b).

### High-speed imaging of cavitation bubbles

These investigations were performed at the Hydraulic Machines Laboratory of the École Polytechnique Fédérale de Lausanne (Lausanne, Switzerland). The tip of the 6-mm applicator of the Swiss DolorClast, mounted on the “radial” handpiece, was submerged in de-ionized water contained within a custom-built transparent cubic vessel made of clear high-density polycarbonate (20 cm side length, 1 cm wall thickness). The DolorClast was operated at 4.0 bar and 5 Hz. Measurements were run in triplicates.

For documentation of cavitation bubble dynamics, a high-speed CCD camera (Photron Ultima APX; Photron, Tokyo, Japan) with a framing rate of 300,000 frames per second and exposure time of 1/2,700,000 seconds was used. Each captured frame comprised a total of 8192 (64 × 128) pixels, encompassing an area of approximately 8.8 × 17.6 mm. Transmitted light illumination was provided by a high-power light source (Cordin Light Source Model 359; Cordin, Salt Lake City, UT, USA). Gas bubbles appeared dark in this setup due to their light scattering properties.

The applicator was lowered from above into the camera frame's top section. Camera recordings were triggered manually prior to the release of a single pulse. Individual film sequences (approximately 10,000 continuous frames, equivalent to film duration of 33 ms) were subsequently visualized using FASTCAM viewer software (Photron, Tokyo, Japan), converted into individual images with 256 greyscales (with zero and 256 representing black and white, respectively), and exported as TIF files (Fig. 5c).

## Author Contributions

M.C.K. S.M., H.G.F. and C.S. designed the study. R.K. supervised the generation of fertilized chicken eggs. M.C.K. and S.M. exposed chicken embryos to radial extracorporeal shock waves. M.C.K. and C.S. performed the analysis and prepared the figures. M.C.K. S.M., H.G.F., R.K. and C.S. wrote the manuscript.

## Figures and Tables

**Figure 1:**
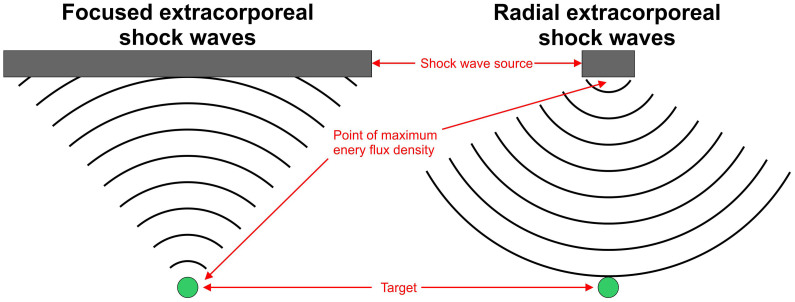
Main differences between focused and radial extracorporeal shock waves. Details are provided in the main text.
